# Perceived Noise Sources and Their Association with Nurses’ Health and Work Performance in Intensive Care Units: A Multicenter Study

**DOI:** 10.3390/healthcare13212790

**Published:** 2025-11-03

**Authors:** Biljana Filipović, Tea Bernardić, Snježana Čukljek, Adriano Friganović, Danijela Kundrata, Sanja Ledinski

**Affiliations:** 1 Department of Nursing, University of Applied Health Sciences, 10000 Zagreb, Croatia; snjezana.cukljek@zvu.hr (S.Č.); adriano@hdmsarist.hr (A.F.); sanja.ledinski@zvu.hr (S.L.); 2 Department of Nursing, Faculty of Health Studies, University of Rijeka, 51000 Rijeka, Croatia; 3 Department of Anesthesiology, Reanimatology and Intensive Care, University Hospital Centre Osijek, 31000 Osijek, Croatia; bernardict555@gmail.com; 4 Department of Quality Assurance and Health Care Improvement, University Hospital Centre Zagreb, 10000 Zagreb, Croatia; 5 Department of Quality Assurance and Health Care Improvement, General Hospital “Dr. Ivo Pedišić”, 44000 Sisak, Croatia; danijela.kundrata@gmail.com

**Keywords:** intensive care unit, noise, nurses, work performance

## Abstract

Background: Modern healthcare environments expose staff to various occupational stressors, with noise being among the most common and harmful stressors. In intensive care units (ICUs), both patients and nurses are frequently exposed to unsafe noise levels, which can adversely affect well-being, recovery, and work performance. Objective: This study aimed to identify sources of noise and their adverse effects from the perspective of ICU nurses, and to examine associations between noise perception, demographic and job-related variables, and outcomes across four domains: subjective, emotional, physiological, and work performance. Methods: A cross-sectional study was conducted from February to September 2023 among 100 ICU nurses employed in three Croatian hospitals: Zagreb, Pula and Slavonski Brod. Data were collected using a validated three-part questionnaire and analyzed with descriptive and inferential statistics. Results: Nurses reported that monitor and ventilator alarms were the most prominent sources of internal noise. Noise perception within and outside ICUs differed significantly depending on the type of institution, ICU, and number of beds (*p* < 0.05). Significant relationships were found between noise exposure and outcomes across all four domains. Conclusions: Noise in ICUs poses a health risk to both patients and nurses, impairing well-being and work performance. Implementing effective noise reduction strategies should be prioritized in critical care settings.

## 1. Introduction

Noise is commonly defined as unwanted or disruptive sound, while noise pollution refers to environmental sound levels perceived as intrusive and disturbing [1]. In hospital settings, elevated noise arises from multiple internal and external sources, including monitor alarms, overhead speakers, slamming doors, and medical equipment [2]. Such exposures have been associated with impaired well-being, disrupted social interactions and communication, anxiety and irritability, and alterations in vital signs such as heart rate, blood pressure, and respiratory rate [3]. This study focuses on intensive care unit (ICU) nurses. Although patients experience adverse noise effects—including sleep fragmentation, delirium risk, and impeded recovery—nurses are the primary, continuous occupants of the ICU soundscape and may be affected through increased stress, cognitive load, communication barriers, and physiological arousal [3,4,5,6,7,8,9]. Accordingly, this study considers noise as both a clinical concern (for patients) and an occupational concern (for nurses) and investigates nurse-centred outcomes.

From a physical acoustics perspective, noise is typically quantified in decibels (dB), a logarithmic scale of sound pressure. An increase of 10 dB is perceived as approximately twice as loud, and an increase of 3 dB corresponds to a doubling of sound intensity [9]. Measurements frequently employ A-weighting to approximate human auditory sensitivity, reported as dB(A) [10]. In ICUs, average levels commonly range from 60 to 70 dB(A), with peak levels exceeding 90 dB(A); notably, several studies indicate that nighttime levels do not decline substantially [9]. These soundscapes reflect the confluence of clinical technologies, multipatient bays, and continuous monitoring requirements that characterize contemporary critical care.

Hospital noise stems from both human activity and equipment [11]. Reported measurements span from neonatal ICUs (minimum of ≈52.5 dB) to other departments with substantially higher maxima, illustrating broad intra-hospital variability [12]. Notably, environmental agencies have suggested hospital sound limits of approximately 45 dB by day and 35 dB by night [13]. Alarm systems further complicate the acoustic environment: an estimated 80–90% of alarms are clinically nonactionable [14], and although volumes can be adjusted, routine practice often prevails over optimization of alarm audibility [15]. A previous study documented ICU noise levels in the 80–90 dB range, with some of the highest contributions attributed to conversations in staff areas (≈89 dB) and device alarms (≈69 dB for ventilator alarms) [16]. Architectural and engineering approaches—such as greater use of sound-attenuating materials and quieter device/alert designs—have been advocated to curb exposure [17]. Despite international recommendations to keep inpatient sound as low as reasonably achievable to protect rest and recovery, these targets remain difficult to achieve in practice.

The implications for healthcare workers are substantial. In ICUs, where nurses spend extended time at the bedside while managing multiple, simultaneous demands—noise has been linked to agitation, irritability, fatigue, stress, and symptoms consistent with professional burnout [18]. Noise is also a barrier to clinical work quality. Evidence from operating rooms shows that ambient noise can impair cognition and work performance: for example, short-term and working memory among anaesthesia trainees was significantly lower during exposure to operating-room noise compared with quieter conditions [19,20]. Outside healthcare, ambient noise similarly exerts detrimental effects on individuals, and its impact depends not only on acoustic characteristics (e.g., intensity, frequency content) but also on non-acoustic features such as meaning and context [21].

ICU noise disrupts sleep by fragmenting rest and reducing slow-wave and REM phases, changes that impair cognition and increase delirium risk, ultimately hindering patient recovery [22]. Sympathetic activation in response to noise may elevate heart rate and disturb respiratory muscle function; excessive noise can increase sedative needs, hinder communication, and contribute to hearing problems, further amplifying vulnerability in the critically ill. These patient-centred concerns coexist with the occupational health burden borne by staff, creating a dual imperative to address noise as both a clinical and organizational priority.

Despite numerous measurements and interventions reported in the literature, multicentre evidence focusing on nurses’ perceptions of ICU noise—and how these perceptions relate to health and work performance—remains limited. Perception is a crucial mediator between exposure and outcome: it shapes emotional and physiological responses and informs behavioural adaptations in complex, time-critical environments. Moreover, demographic and job-related variables—such as unit type (e.g., coronary vs. polyvalent), number of beds, shift patterns, and institutional characteristics—may systematically modulate how noise is perceived and the extent to which it affects subjective, emotional, physiological, and work-performance domains. This is consistent with prior studies emphasizing nurse- or patient-reported perceptions of noise and their impact on communication and work performance [9,14,15,18,19].

To address this gap, this multicentre study examines perceived noise sources and their associations with ICU nurses’ health and work performance. Specifically, it identifies internal and external noise sources from the perspective of ICU nurses, assesses differences in noise perception within and outside ICUs across demographic and job-related variables, and investigates associations between perceived noise and outcomes in four domains: subjective, emotional, physiological, and work performance.

## 2. Materials and Methods

### 2.1. Study Design

This research was conducted as a multicenter cross-sectional study. The study was conducted between 1 February and 1 September 2023, in three hospitals in Croatia: Zagreb, Pula and Slavonski Brod.

### 2.2. Participants

Completed questionnaires were collected from 40 nurses from General Hospital Pula (out of 55 employed in ICUs; 72.7% response rate), 35 from Clinical Hospital Merkur, Zagreb (out of 45; 77.8%), and 25 from General Hospital “Dr. Josip Benčević”, Slavonski Brod (out of 45; 55.6%).

### 2.3. Selection Criteria

Inclusion criteria were registered nurses working in ICUs, regardless of years of professional experience.

Exclusion criteria were nurses who did not provide informed consent or submitted incomplete questionnaires.

### 2.4. Procedure

Paper questionnaires were distributed by the unit head nurses, who were briefed by the research team. A sealed-envelope procedure was used: completed questionnaires were placed in unmarked envelopes and deposited in a locked collection box on each unit; no names or employee identifiers were collected. Only aggregated, de-identified data were analyzed. Information about the study purpose and procedures was provided in writing by the research team and verbally by the head nurse at each shift handover. Participation was voluntary and uncompensated.

### 2.5. Instrument

Data were collected using a questionnaire developed by Kooshanfar et al. [23], applied with permission. The instrument consisted of three sections:

1. Personal and professional characteristics of respondents.
2. Assessment of internal and external sources of noise: 11 items evaluated internal noise (within the ICU), and 9 items evaluated external noise (outside the ICU but within or outside the hospital). Each item was rated according to perceived degree of impact (small, moderate, large).
3. Impact of noise across four domains: subjective perception (anxiety, inattention, insomnia, fear, dizziness), emotional impact (communication problems, irritability, tension, loss of patience, tingling sensations), physiological effects (tachycardia, tinnitus, headache, fatigue, loss of appetite), and work performance (reduced productivity, concentration problems, negligence, disobedience, auditory masking).

All items were assessed on a five-point Likert scale (1 = never, 2 = rarely, 3 = sometimes, 4 = often, 5 = always). A forward-backward translation was performed by two independent professional translators. An expert panel of Croatian ICU nursing professionals (*n* = 5) evaluated semantic, idiomatic, experiential, and conceptual equivalence. Each modification was agreed by consensus to preserve the original constructs. A pilot test with *n* = 12 ICU nurses from a non-participating unit assessed clarity and completion time and no structural changes were required. Internal consistency in the main sample was high (Cronbach’s α = 0.95).

### 2.6. Analysis

Descriptive statistics were used to summarize the sample characteristics. Continuous variables are reported as means with standard deviations (M ± SD), and categorical variables as absolute and relative frequencies (n, %). Age was categorized into four predefined groups: 18–25, 26–35, 36–45, and ≥46 years. Normality of distribution was assessed using the Kolmogorov–Smirnov test, and homogeneity of variances using Levene’s test. Where assumptions were met, parametric tests were used: independent *t*-test for two-group comparisons and one-way ANOVA for comparisons involving three or more groups. When assumptions were violated, non-parametric alternatives were applied: the Mann–Whitney U test for two groups and the Kruskal–Wallis H test for three or more. Post hoc analyses included Tukey’s test after ANOVA and Dunn’s test after Kruskal–Wallis, with adjusted *p*-values where applicable. Associations between internal and external noise exposure (as continuous scale scores) and each of the four outcome domains (subjective perception, emotional response, physiological symptoms, and work performance) were examined using Spearman’s rank correlation coefficients (ρ), due to non-normality in several distributions. All statistical tests were two-tailed, with significance set at *p* < 0.05. Analyses were conducted using IBM SPSS Statistics for Windows, Version 25.0 (IBM Corp., Armonk, NY, USA) and JASP, Version 0.17.2.1 (University of Amsterdam, Amsterdam, The Netherlands).

### 2.7. Ethics

The study was approved by the Ethics Committees of General Hospital Pula (Class: 641-01/23-01/01, Approval No. 2168/01-59-79-112-20-21, dated 20 March 2023.), Clinical Hospital Merkur (Approval No. 03/1-2995, dated 19 April 2023.), and General Hospital “Dr. Josip Benčević” Slavonski Brod (dated 24 February 2023.). Written informed consent was obtained from all nurses prior to their involvement. Participation was voluntary, anonymity and confidentiality were guaranteed, and nurses were informed of their right to withdraw from the study at any point without consequence. Permission to use the questionnaire was obtained from the original author.

## 3. Results

A total of 100 ICU nurses participated in the study. The majority were women (*n* = 87; 87%). The largest age group was 36–45 years (*n* = 39; 39%), and most nurses were married (*n* = 87; 87%). Regarding education, 46 nurses (46%) had completed secondary nursing school, while others held higher nursing qualifications. With respect to workplace distribution, most nurses were employed in polyvalent ICUs (*n* = 69, 69%), followed by coronary care ICUs (*n* = 31, 31%). The mean age of nurses was 36.45 years (range: 20–62 years). Detailed demographic and professional characteristics are presented in Table 1.

Nurses most frequently identified monitor and ventilator alarms as the primary sources of noise (M = 2.56, SD = 0.64), while the lowest agreement was for “Sound of radio and television” (M = 1.33, SD = 0.49). For external sources, the highest scores were reported for “Hospital renovation and other construction work” (M = 1.95, SD = 0.74), whereas the lowest were again for “Sound of radio and television” (M = 1.22, SD = 0.48) (Table 2).

Table 3 presents the overall results of the noise scales within and outside ICUs, as well as the mean values for subjective, emotional, physiological, and work performance domains. The highest mean score was recorded for the physiological domain (M = 12.99, SD = 4.31; range 5–21), followed by the emotional domain (M = 11.36, SD = 3.75; range 5–23) (Table 3). Fatigue, headache, irritability, and tension were the most frequently reported symptoms. The subjective domain had a lower mean score (M = 10.54, SD = 3.76; range: 5–18) (Table 3) indicating moderate experiences of anxiety, inattention, and insomnia. The lowest impact was observed in the work performance domain (M = 9.30, SD = 3.26; range: 5–19), although nurses still reported reduced productivity and concentration.

With respect to noise scales, the mean value for internal ICU noise was higher (M = 20.37, SD = 4.02; range: 12–28) than for external noise (M = 14.95, SD = 3.86; range: 9–23), suggesting that equipment and alarms within ICUs were perceived as more disturbing than external environmental factors.

The results indicated a significant difference in the perception of internal ICU noise according to institution type (Mann–Whitney test, *p* = 0.006) and workplace setting (Mann–Whitney test, *p* = 0.006). Nurses employed in clinical hospitals and those working in coronary care units perceived significantly higher levels of internal noise. A significant difference was also observed with respect to the number of beds (Kruskal–Wallis test, *p* = 0.017). Post hoc Dunn comparisons revealed that nurses working in ICUs with six beds reported significantly higher noise levels compared to those working in ICUs with nine beds (*p* = 0.036) (Table 4).

The results showed a significant difference in the perception of external ICU noise according to institution type (*t*-test, *p* = 0.012) and workplace setting (*t*-test, *p* = 0.012). Nurses employed in clinical hospitals and those working in coronary care units perceived significantly higher levels of external noise. A significant difference was also found with respect to the number of beds (one-way ANOVA, *p* = 0.008). Post hoc Tukey comparisons revealed that nurses working in ICU with six beds reported significantly higher noise levels compared to those working in ICU with twelve beds (*p* = 0.006) (Table 4).

Internal ICU noise was positively correlated with all four domains: subjective (*p* < 0.001), emotional (*p* < 0.001), physiological (*p* < 0.001), and work performance (*p* = 0.001). A strong positive correlation was also found between internal and external noise (*p* < 0.001). External ICU noise showed moderate positive correlations with subjective perception (*p* < 0.001), emotional perception (*p* = 0.001), and physiological perception (*p* < 0.001), as well as a weak positive correlation with work performance (*p* = 0.006). Subjective perception was strongly correlated with emotional and physiological perception (*p* < 0.001 for each) and moderately with work performance (*p* < 0.001). Emotional perception was strongly correlated with physiological perception and work performance (*p* < 0.001 for both). Physiological perception showed a strong positive correlation with work performance (*p* < 0.001) (Table 5).

## 4. Discussion

Our findings extend prior ICU acoustics work by showing that alarm-dominated soundscapes are associated with nurse-reported symptoms across subjective, emotional, physiological, and performance domains. This interpretation is consistent with the observed associations between internal ICU noise and outcomes across all four domains (Table 5). Rather than reiterating descriptive distributions, we emphasize plausible mechanisms—most notably cognitive load and reduced speech intelligibility (as reflected by nurses’ reports of communication problems and auditory masking in the work performance domain) as pathways linking noise with reduced concentration and productivity. Workload and staffing may modulate noise perception and its impact. Higher patient-to-nurse ratios and surges in task demands can amplify alarm burden and conversational traffic, increasing perceived noise and strain [24,25,26]. Although we did not directly measure staffing ratios or patient acuity, these factors warrant consideration in future studies and in multicomponent interventions (alarm governance, workflow redesign, and architectural mitigation).

The identification of alarms as the most prominent internal noise source aligns with international evidence indicating that alarm systems in ICUs are often excessive and clinically nonactionable, with estimates suggesting that 80–90% of alarms do not require intervention [14]. This phenomenon, often referred to as alarm fatigue, not only undermines staff performance but also compromises patient safety. The present multicentre Croatian study contributes further evidence, demonstrating that nurses perceive alarms as a leading contributor to stress and cognitive distraction during routine clinical work. External noise, primarily stemming from renovation and construction activities, was also perceived as highly disruptive. These findings underscore the importance of hospital-level planning, as infrastructural changes and maintenance may intensify acoustic strain in already demanding ICU environments. Built environment factors such as spatial layout, material selection, and ceiling height can greatly influence acoustic conditions in ICUs. These elements are increasingly recognized as critical determinants of staff well-being and concentration. In addition, several aspects of the built environment, such as poor acoustic insulation, reflective surfaces, and lack of sound-absorbing materials, can intensify perceived noise and contribute to stress and cognitive fatigue among ICU nurses.

Differences in noise perception were associated with the type of institution, unit, and number of beds. Nurses employed in clinical hospitals and those working in coronary care units reported significantly higher perceived noise levels. This can be explained by the fact that coronary ICUs require continuous monitoring of high-acuity patients, resulting in more frequent alarms, greater staff activity, and higher ambient noise levels. Previous studies have also emphasized that unit size and organizational factors significantly modulate noise levels. For example, Jung et al. reported considerable variability in ICU noise exposure across different institutional settings, highlighting the role of workload intensity and structural conditions [26]. Likewise, Darbyshire and Young found that measured noise levels varied between UK ICUs and were strongly affected by spatial layout and the arrangement of equipment around patients [27]. Consistent with this, Nyembwe et al. observed that structural and spatial constraints contribute to suboptimal acoustic environments, with limited noise insulation increasing perceived noise among staff [28]. In our study, nurses working in six-bed ICUs perceived significantly higher levels of noise compared with those in nine- or twelve-bed units, which supports the argument that structural and organizational differences strongly influence the acoustic environment.

Associations between noise perception and subjective, emotional, physiological, and performance-related outcomes further support evidence linking environmental noise with occupational health. Subjective symptoms such as insomnia and disturbed sleep continuity were significantly associated with higher perceived noise, echoing prior findings that noise contributes to psychological strain and sleep disturbances [29]. Emotional outcomes including irritability, communication problems, and tension were also strongly correlated with noise exposure, which is consistent with studies identifying noise as a barrier to teamwork and a driver of burnout in nurses [30]. Physiological symptoms such as tachycardia, headache, and fatigue—captured within the physiological domain—were frequently reported, aligning with literature on the activation of the sympathetic nervous system in response to noise [31]. Work performance was negatively affected by noise, particularly through reduced concentration and productivity. Similar findings have been reported in operating room environments, where background noise has been shown to impair memory and task accuracy among healthcare professionals [32].

### 4.1. Implications for Nursing Practice

These findings have several implications for nursing practice and healthcare system management. Priority should be given to alarm management. The implementation of smart alarm systems, threshold customization, and targeted staff training may mitigate alarm fatigue, thereby improving both staff well-being and patient safety. Architectural and organizational strategies—such as the use of sound-absorbing materials, optimized room layouts, and workflow adjustments—could reduce overall noise exposure. Hospital administrators should take noise into account during renovation planning and ensure that ICUs are adequately shielded from construction-related disturbances. Furthermore, maintaining adequate nurse staffing levels and implementing balanced scheduling may reduce cumulative noise-related stress, particularly for nurses working night or rotating shifts. Some of these strategies, such as alarm management protocols and architectural noise mitigation, have been successfully piloted in ICU settings. For example, a recent implementation study demonstrated sustained reduction in sound levels using a multi-component noise management bundle [33], while a systematic review highlighted improved staff satisfaction and patient outcomes following environmental modifications like acoustic insulation [34]. Future research and practice could also benefit from structured tools such as the Ward Assessment Instrument developed by Durosaiye et al., which offers a multidimensional approach to evaluating built environmental conditions in clinical settings [35].

### 4.2. Strengths of the Study

The strengths of this study include its multicenter design, which increases the diversity and representativeness of the sample, and the use of a validated instrument adapted to the Croatian nursing context. The large set of variables analyzed allowed for a comprehensive exploration of how noise perception interacts with demographic and job-related factors, as well as with health and performance outcomes.

### 4.3. Limitations of the Study

Noise was assessed through self-report rather than objective measurements. While perception is essential for understanding the impact of noise on well-being and behavior, combining subjective data with decibel measurements would provide a more complete picture. The cross-sectional design limits causal inference; associations were observed, but causal direction cannot be firmly established. The convenience sampling from three Croatian hospitals limits generalizability beyond similar ICU settings. Perceptions were measured via self-report, which is susceptible to recall and social-desirability biases; pairing surveys with objective dosimetry (dB[A]) and staffing data would strengthen inference. Also, unmeasured confounders such as patient acuity, staffing ratios, and workload intensity may have influenced the observed associations.

Future research should integrate objective noise measurements with self-reported data, employ longitudinal designs to establish causal links, and test targeted interventions for noise reduction.

## 5. Conclusions

This multicentre study underscores that noise in intensive care units remains a critical occupational and organizational challenge. Monitor and ventilator alarms were identified as the most disruptive internal noise sources, while renovation and construction activities were perceived as the dominant external contributors. Nurses employed in clinical hospitals and those working in coronary care units reported higher levels of noise perception. Notably, smaller ICU size was associated with greater perceived noise; nurses in six-bed units reported significantly higher levels compared with those in larger units. Both internal and external noise were significantly associated with outcomes across subjective, emotional, physiological, and work-performance domains, indicating adverse effects on nurses’ well-being and clinical functioning.

These findings emphasize that ICU noise is not merely a background issue but a systemic factor influencing both staff functioning and patient care. To effectively address this problem, hospitals must adopt a holistic noise management strategy that includes organizational awareness, environmental modifications, and alarm governance. Implementing these aspects in daily practice may reduce occupational stress, improve communication, and enhance staff performance in high-demand clinical settings.

## Figures and Tables

**Table 1:** Demographic and professional characteristics of ICU nurses (N = 100).

		Number (%) of Nurses
Sex	Male	13 (13)
	Female	87 (87)
Age M = 36.45 (SD = 10.44)	18–25	21 (21)
	26–35	23 (23)
	36–45	39 (39)
	46 and older	17 (17)
Marital status	Single	13 (13)
	Married	87 (87)
Education	Secondary nursing school	46 (46)
	Bachelor of Nursing	41 (41)
	Master of Nursing	13 (13)
Work shifts	Morning/afternoon shift	13 (13)
	Shift work	57 (57)
	Both (morning/afternoon + rotating shifts)	30 (30)
Type of institution	Clinical Hospital Center (CHC)	0
	Clinical Hospital (CH)	31 (31)
	General Hospital (GH)	69 (69)
Department of employment	Polyvalent ICU	69 (69)
	Coronary Care ICU	31 (31)
Number of beds	6	31 (31)
	9	29 (29)
	12	40 (40)
Job title	Head nurse	3 (3)
	Ward nurse	97 (97)
Years of service *	<5	24 (24)
	5–14 years	29 (29)
	15–24 years	33 (33)
	>25 years	14 (14)
Work experience in ICU *	<5	32 (32)
	5–13 years	36 (36)
	>13 years	32 (32)
Overtime hours	<10 h	65 (65)
	>10 h	25 (25)

ICU = intensive care unit; CH = clinical hospital; GH = general hospital; * “Years of service” refers to total professional experience as a nurse, while “ICU experience” refers to years worked specifically in intensive care settings.

**Table 2:** Perceived internal and external sources of noise in intensive care units.

	1 = Little	2 = Moderate	3 = Much		
	Number (%) of Nurses			M (Range)	SD
Internal ICU noise					
Monitor and ventilator alarms	8 (8)	28 (28)	64 (64)	2.56 (1–3)	0.64
Telephone ringing	7 (7)	34 (34)	59 (59)	2.52 (1–3)	0.62
Patient expressing pain	28 (28)	43 (43)	29 (29)	2.01 (1–3)	0.75
Conversation between healthcare staff	28 (28)	55 (55)	17 (17)	1.89 (1–3)	0.66
Oxygen and suction devices	32 (32)	37 (37)	31 (31)	1.99 (1–3)	0.79
Medical equipment	32 (32)	34 (34)	34 (34)	2.02 (1–3)	0.81
Conversation between visitors or family members	51 (51)	39 (39)	10 (10)	1.59 (1–3)	0.66
Opening and closing doors	61 (61)	16 (16)	23 (23)	1.62 (1–3)	0.83
Sound of radio and television	68 (68)	31 (31)	1 (1)	1.33 (1–3)	0.49
Opening drawers or wardrobes	70 (70)	24 (24)	6 (6)	1.36 (1–3)	0.59
Room cleaning	62 (62)	28 (28)	10 (10)	1.48 (1–3)	0.67
External ICU noise					
Hospital renovation and other construction work	30 (30)	45 (45)	25 (25)	1.95 (1–3)	0.74
Patient admission	37 (37)	48 (48)	15 (15)	1.78 (1–3)	0.69
Patient expressing pain	51 (51)	31 (31)	18 (18)	1.67 (1–3)	0.76
Conversation between healthcare staff	37 (37)	40 (40)	23 (23)	1.86 (1–3)	0.76
Conversation between visitors or family members	62 (62)	30 (30)	8 (8)	1.46 (1–3)	0.64
Rolling of trolley wheels	41 (41)	33 (33)	26 (26)	1.85 (1–3)	0.80
Opening and closing doors	54 (54)	29 (29)	17 (17)	1.63 (1–3)	0.76
Hospital cleaning	56 (56)	35 (35)	9 (9)	1.53 (1–3)	0.65
Sound of radio and television	81 (81)	16 (16)	3 (3)	1.22 (1–3)	0.48

ICU = intensive care unit; M = mean; SD = standard deviation.

**Table 3:** Mean values of noise perception across four domains: subjective, emotional, physiological, and work performance.

	M (Range)	SD
Internal ICU noise	20.37 (12–28)	4.02
External ICU noise	14.95 (9–23)	3.86
Subjective perception	10.54 (5–18)	3.76
Emotional perception	11.36 (5–23)	3.75
Physiological perception	12.99 (5–21)	4.31
Work performance	9.30 (5–19)	3.25

ICU = intensive care unit; M = mean; SD = standard deviation.

**Table 4:** Perceived internal and external ICU noise by job-related variables.

		Internal Noise	External Noise	Internal Noise	External Noise	Internal Noise	External Noise
		M (Range)		SD		*p*	
Work shifts	Morning/afternoon shift	19.30 (12–27)	14.15 (9–20)	4.269	3.43	0.485 *	0.230^‡^
	Shift work	20.85 (12–28)	15.52 (9–23)	4.48	4.11		
	Both (morning/afternoon + rotating shifts)	19.90 (12–24)	14.20 (10–21)	2.79	3.43		
Type of institution	Clinical Hospital (CH)	22.25 (14–28)	16.38 (8–23)	4.66	3.63	0.006 **	0.012 ^†^
	General Hospital (GH)	19.52 (12–27)	14.30 (9–23)	3.41	3.81		
Department of employment	Polyvalent ICU	19.52 (12–27)	14.30 (9–23)	3.41	3.81	0.006 **	0.012 ^†^
	Coronary Care ICU	22.25 (14–28)	16.38 (8–23)	4.66	3.63		
Number of beds	6	22.25 (14–28)	16.38 (9–23)	4.66	3.63	0.017 *	0.008 ^‡^
	9	19.20 (12–27)	15.27 (9–23)	4.19	4.07		
	12	19.75 (12–24)	13.60 (9–21)	2.76	3.49		
Job title	Head nurse	19.66 (13–28)	14.33 (10–19)	7.63	4.50	0.761 ^†^	0.780 ^†^
	Ward nurse	20.39 (12–28)	14.96 (9–23)	3.93	3.86		
Years of service	<5 years	20.33 (12–28)	15.00 (9–23)	3.93	3.67	0.900 ^‡^	0.723 ^‡^
	5–14 years	20.72 (14–27)	15.48 (9–21)	4.01	3.87		
	15–24 years	20.36 (12–28)	14.36 (9–20)	4.30	3.62		
	>25 years	19.71 (12–26)	15.14 (9–23)	3.89	4.83		
Work experience in ICU	<5 years	19.84 (12–28)	14.50 (9–23)	4.05	3.61	0.309 ^‡^	0.106 ^‡^
	5–13 years	21.19 (14–28)	16.02 (9–21)	3.83	3.76		
	>13 years	19.96 (12–28)	14.18 (9–23)	4.18	4.05		
Overtime hours	<10 h	20.30 (12–28)	15.13 (9–23)	3.88	3.65	0.834 ^†^	0.396 **
	>10 h	20.48 (12–28)	14.60 (9–21)	4.33	4.25		

* Kruskal–Wallis test; ** Mann–Whitney test; ^†^ *t*-test; ^‡^ One-way ANOVA; ICU = intensive care unit; M = mean; SD = standard deviation.

**Table 5:** Correlations between noise perception and subjective, emotional, physiological, and work performance outcomes.

		1.	2	3	4	5	6
Internal ICU noise	rho	1					
	*p* *						
External ICU noise	rho	0.634					
	*p* *	<0.001					
Subjective perception	rho	0.485	0.460				
	*p* *	<0.001	<0.001				
Emotional perception	rho	0.448	0.335	0.557			
	*p* *	<0.001	0.001	<0.001			
Physiological perception	rho	0.575	0.367	0.730	0.754		
	*p* *	<0.001	<0.001	<0.001	<0.001		
Work performance	rho	0.326	0.273	0.482	0.686	0.564	1
	*p* *	0.001	0.006	<0.001	<0.001	<0.001	

rho—Spearman’s correlation coefficient; *p*—statistical significance; * Spearman’s correlations; ICU = intensive care unit.

## Data Availability

Study data are available upon request from the corresponding author.
